# Supplementary material for: A multi-mechanism approach reduces length of stay in the ICU for severe COVID-19 patients
Source: PLoS One. 2021 Jan 7;16(1):e0245025. doi: 10.1371/journal.pone.0245025 (PMC7790264; doi:10.1371/journal.pone.0245025)
Supplement: S1 File — (DOCX) [file pone.0245025.s001.docx]

# **Supporting information**

## **Supplemental information on rationale for Multi Mechanism Approach**

At the time of this study, no specific antiviral agent has demonstrated a definitive mortality benefit. Hence, in our study there were no preference for which antiviral regimen was used (**shown in S1 Table**). Our available options included any of the antiviral medications used in the SOLIDARITY clinical trial [31]. Although no antiviral agents have demonstrated significant benefit, other pharmacological agents may have potential benefits. Ivermectin is widely available and inhibits the importin channel (IMPα/β1) [32]. Recent studies under in-vitro conditions revealed that it can reduce the virus up to 5000-fold in culture [33]. Azithromycin (AZ) has shown therapeutic effects, acting as an acidotropic lipophilic weak base which modulate the pH of endosomes and Golgi network [34]. The use of experimental antivirals is still controversial. Due to the retrospective nature of this study and non-randomization of the participants, we cannot establish a direct relationship between the individual drugs and the outcome of the participants.

**S1 Table. Antivirals available during the study time**

| **Classification of Treatment** | **Medication** | **Dosage** | **Frequency** | **Route of Administration** | **Duration of Treatment** |
| --- | --- | --- | --- | --- | --- |
| Antivirals | Hydroxychloroquine | 400mg initial dose, 200mg subsequent doses | every 12 hours | Oral | 5 days |
|  | Ivermectin | 200 mcg/kg/day | every day | Oral | 5 days |
|  | Azithromycin | 500mg | every day | Oral | 5 days |

With the MMA treatment, we were targeting the immune response, which has been highlighted as a pivotal factor in terms of mortality and complications. The use double anti-inflammatory agents (corticosteroids and colchicine) to anticipate the unpredictable cytokine release syndrome observed in COVID-19 patients. All this data is supported by recent studies, published by Oxford University in which they found dexamethasone reduced deaths by one third in ventilated patients (HR 0.65, 95% CI 0.48-0.88, p-value 0.0003) [35]. In the GRECCO-19 study, it was found that the participants who received colchicine had statically significant improved time to clinical deterioration (event-free survival time was 18.6 days in the control group versus 20.7 in the colchicine group, Log-rank P: 0.03) [6]. However, in some patients, this anti-inflammatory regimen may be insufficient to control the inflammatory state at the time of presentation to the hospital. Thus, rescue agent (Tocilizumab) may be required, in the TESEO cohort found benefits in overall mortality and a risk reduction of the use of mechanical ventilation [26].

Several studies, reported the severity of the disease has strong correlation with elevated coagulation markers, such as DD and FDP. Thus, we can state that SARS-Cov2 can activate the coagulation cascade inducing a procoagulant state [10]. Additionally, the cytochemistry studies reveal deposits of VWF indicating platelet activation that further contributes to the formation of thrombi. Moreover, studies show complement deposits supporting the inflammatory response as an immune thrombosis [36]. There are ongoing discussions on whether heparin and low molecular weight heparin reduce mortality as well as halting progression to the more severe stages of the disease. Among them the best known is its anticoagulant property is heparin, it also has anti-inflammatory properties by inhibiting IL-6 [11]. There has yet to be a final consensus reached regarding this matter. Some clinical studies support the use of heparin [9-11]. There is an observed mortality reduction with the use of heparin (40% versus 64% without heparin, P-value 0.02), in our study patients using standard care with 40% mortality we found D-D levels higher (2.3±2.6, p-value 0.004) at the time of admission to ICU when compared with MMA, which may be due that patients with standard care did not receive a full dose of anticoagulants at the time of admission to the hospital.

The management of respiratory failure secondary to SARS-Cov-2 infection has been a challenge for health personnel, and experience in managing these patients with invasive mechanical ventilation has shown negative outcomes with mortality up to 88% of the patients. Avoiding endotracheal intubation may benefit COVID-19 patients to achieve survival by providing a bridge to therapies as corticosteroids, immunomodulation, anticoagulation, and inhibitors of IL6 in the most severe cases to work while supplying adequate oxygenation. High-Flow nasal cannula (HFNC) has shown a greater benefit than conventional noninvasive oxygen therapies in two studies; In the FLORALI trial [37], patients with non-hypercapnic acute hypoxemic respiratory failure treatment with high-flow oxygen, they found significant difference in 90 day mortality. In a post-hoc analysis [38], it involved 82 immunocompromised patients with severe respiratory failure, using HFNC 31% required intubation, compared with conventional oxygen therapy (COT) and non-invasive ventilation (NIV) 43% and 65% required intubation respectively. Similar results were obtained by Coudroy et al in an observational cohort study [36] were HFNC required fewer intubations than NIV (35 vs 55%) and lower 28 days mortality with HFNC (20 vs 40%). During this SARS-CoV-2 pandemic, patients experiencing hypoxic respiratory insufficiency in a few small single-center studies have reported significant improvement in awake non intubated patients. In a group of 24 patients with a respiratory rate greater than 30 per min and saturation less than 93%, they were prone for one hour, observing improvement in saturation up to 95% and less intubation. The benefit of this strategy was more profound when prone positioning was achieved for 16 hrs [12, 34-36]. In our study, we observed that in patients that used high flow systems had better outcomes, where 83% survived (p-value 0.01).

This study explores the effectiveness of a timely initiation of a combination of immunomodulators (tocilizumab and colchicine), anti-inflammatory drugs (glucocorticoids), therapeutic anticoagulation, experimental antivirals (HCQ-AZ, and Ivermectin) and to provide adequate alternative ventilatory support other than mechanical ventilation to patients with hospitalized COVID – 19 disease.

**Supplementary Tables**

**S2 Table. Characteristics between Survived and non- survived patients**

| **Characteristics** | **Survived**  **N= 48**  **(%)** | **Non-Survived**  **N= 17**  **(%)** | **P-value** | **Total**  **N=65** | **Range**  **(Min-Max)** |
| --- | --- | --- | --- | --- | --- |
| **Age (years)** | 51.6±16.1 | 60.4±15.6 | 0.05 | 53.9±16.4 | (18-86) |
| **Treatment group** | | | | | |
| **S.C** | 18 (38) | 12 (70) |  | 30 (46) | - |
| **MMA** | 30 (62) | 5 (29) | 0.01 | 35 (54) | - |
| **Comorbidities** | | | | | |
| <2 | 25 (52) | 9 (53) | 0.49 | 34(52) | - |
| >2 | 15 (31) | 7 (41) |  | 22 (34) | - |
| None | 8 (17) | 1 (6) |  | 9 (14) | - |
| Hypertension | 21 (44) | 12 (70) | 0.05 | 33 (51) | - |
| Diabetes | 15 (31) | 5 (29) | 0.88 | 20(31) | - |
| Obesity | 14 (29) | 8 (47) | 0.23 | 22 (34) | - |
| Thyroid Disease | 2 (4) | 2 (12) | 0.27 | 4 (6) | - |
| Cardiovascular diseases | 4 (8) | 2 (12) | 0.64 | 6 (9) | - |
| Asthma | 3 (6) | 1 (6) | 1.0 | 4 (6) | - |
| Cancer | 2 (4) | 1 (6) | 1.0 | 3 (5) | - |
| Other diseases | 11 (23) | 4 (24) | 1.0 | 15 (23) | - |
| **Gender** | | | | | |
| Female | 14 (29) | 5 (29) | 0.98 | 19 (29) | - |
| Male | 34 (71) | 12 (71) |  | 46 (71) | - |
| **Severity scales** | | | | | |
| **EWS (>7)** | 48 (100) | 17 (100) | - | 65 (100) | - |
| **Quick- SOFA** | | | | | |
| 1 | 41 (85) | 9 (53) | 0.006 | 50 (77) | - |
| 2-3 | 7 (15) | 8 (47) |  | 15 (23) | - |
| **Days from symptoms onset prior to presenting to the hospital** | 6.6±5.3 | 6.8±4.0 | 0.63 | 6.6±5.0 | (1-24) |
| **Laboratory findings at the time of admission to ICU** | | | | | |
| Lymphocyte count | 1131±916 | 1042±905 | 0.47 | 1108±907 | - |
| Ferritin | 1173±1000 | 1917±1551 | 0.12 | 1356±1191 | - |
| LDH | 491±504 | 593±262 | 0.5 | 513±4631 | - |
| D-Dimer | 1.3±1.7 | 1.8±2.4 | 0.27 | 1.49±1.97 | - |
| **Respiratory Support** | | | | | |
| Mechanical Ventilation | 8 (17) | 8 (47) | 0.02 | 16 (25) | - |
| High flow | 40 (83) | 9 (53) | 0.01 | 49 (75) | - |
| *Required mechanical ventilation after High flow* | *3 (6)* | *8 (47)* | *<0.0001* | *11 (17)* | *-* |

*Sociodemographic characteristics, severity scales at the admission to ICU, laboratory findings, respiratory support needed, and treatment group*

**S3 Table. Event versus treatment adjusted analysis**

| **Predictors** | **P-value** | **Regression Coefficient** |
| --- | --- | --- |
| **Mechanical Ventilation** | 0.1 | 1.3 |
| **Q-SOFA** ≥**2** | 0.1 | 1.1 |
| **Gender (male)** | 0.8 | -0.19 |
| **Hypertension** | 0.1 | 1.12 |
| **Treatment Group (MMA)** | 0.6 | -0.40 |

**Q-SOFA (Quick- Sequential Organ Failure Assessment) , MMA (Multi-Mechanism Approach). With no significant coefficients, in terms of mortality MMA failed to reached significance.*

**S4 Table. LOS in ICU versus treatment, adjusted analysis**

| **Predictors** | **P-value** | **Regression Coefficient** |
| --- | --- | --- |
| **Treatment**  **(MMA)** | 0.02 | 0.04 |
| **Age** | 0.8 | -0.0007 |
| **Mechanical Ventilation** | 0.6 | -0.01 |
| **Q-SOFA** ≥ **2** | 0.8 | 0.002 |
| **Hypertension** | 0.8 | -0.002 |
| **Gender (male)** | 0.7 | -0.005 |
| **Event (death)** | 0.8 | -0.003 |

**MMA (Multi-mechanism Approach), Q-SOFA (Quick-Sequential Organ Failure Assessment), LOS in ICU (Length of Stay in Intensive Care Unit). Treatment group the only significant coefficient* .

**S5 Table. Stratification by gender**

| **Characteristics** | **Male**  **N= 46**  **(%)** | **Female**  **N= 19**  **(%)** | **P-value** | **Total**  **N=65** | **Range**  **(Min-Max)** |
| --- | --- | --- | --- | --- | --- |
| Age (years) | 52.3±15.5 | 57.8±18 | 0.17 | 53.9±16.4 | (18-86) |
| Treatment group | | | | | |
| S.C | 22(48) | 8 (42) | 0.67 | 30 (46) | - |
| MMA | 24(52) | 11 (58) | - | 35 (54) | - |
| Comorbidities | | | | | |
| Hypertension | 21 (45) | 12 (63) | 0.19 | 33 (51) | - |
| Diabetes | 10 (22) | 10 (52) | 0.01 | 20(31) | - |
| Cardiovascular diseases | 5 (11) | 1 (5) | 0.47 | 6 (9) | - |
| Severity scales | | | | | |
| EWS (>7) | 46 (100) | 19 (100) | - | 65 (100) | - |
| Quick- SOFA | | | | | |
| 1 | 33(72) | 17 (89) | 0.12 | 50 (77) | - |
| 2-3 | 13 (48) | 2 (11) |  | 15 (23) | - |
| Laboratory findings at the time of admission to ICU | | | | | |
| Lymphocyte count | 1223±1018 | 806 ±386 | 0.30 | 1108±907 | - |
| Ferritin | 1609±2195 | 708±784 | 0.34 | 1356±1191 | - |
| LDH | 576±530 | 356±146 | 0.45 | 513±4631 | - |
| D-Dimer | 1.6±2.1 | 1.1±1.35 | 0.59 | 1.49±1.97 | - |
| Respiratory Support | | | | | |
| Mechanical Ventilation | 15 (33) | 1(5) | 0.02 | 16 (25) | - |
| High flow | 31 (67) | 18 (95) | 0.02 | 49 (75) | - |
| Outcomes | | | | | |
| Survived | 34 (74) | 14 (74) | 0.98 | 48 (74) |  |
| Non-survived | 12 (26) | 5 (26) | - | 17(26) |  |

*Sociodemographic characteristics, severity scales at the admission to ICU, laboratory findings, respiratory support needed, and treatment group.*

# **References**

[31] Public health emergency SOLIDARITY trial of treatments for COVID-19 infection in hospitalized patients n.d. [Internet] http://www.isrctn.com/ISRCTN83971151 (accessed July 19, 2020).

[32] Sharun K, Dhama K, Patel SK, Pathak M, Tiwari R, Singh BR, et al. Ivermectin, a new candidate therapeutic against SARS-CoV-2/COVID-19. Ann Clin Microbiol Antimicrob. 2020 May 30;19(1):23.

[33] Lagier JC, Million M, Gautret P, Colson P, Cortaredona S, et al. Outcomes of 3,737 COVID-19 patients treated with hydroxychloroquine/azithromycin and other regimens in Marseille, France: A retrospective analysis. Travel Med Infect Dis. 2020 Jun 25;36:101791.

[34] Deftereos S, Giannopoulos G, Vrachatis DA, Siasos G, Giotaki SG, Cleman M, et al. Colchicine as a potent anti-inflammatory treatment in COVID-19: can we teach an old dog new tricks? Eur Heart J Cardiovasc Pharmacother. 2020 Jul 1;6(4):255.

[35] Lodigiani C, Iapichino G, Carenzo L, Cecconi M, Ferrazzi P, Sebastian T, et al. Humanitas COVID-19 Task Force. Venous and arterial thromboembolic complications in COVID-19 patients admitted to an academic hospital in Milan, Italy. Thromb Res. 2020 Jul;191:9-14.

[36] Drake MG. High-Flow Nasal Cannula Oxygen in Adults: An Evidence-based Assessment. Ann Am Thorac Soc. 2018 Feb;15(2):145-155.

[37] Frat JP, Ragot S, Girault C, Perbet S, Prat G, Boulain T, et al. Effect of non-invasive oxygenation strategies in immunocompromised patients with severe acute respiratory failure: a post-hoc analysis of a randomised trial. Lancet Respir Med. 2016 Aug;4(8):646-652.

[38] Coudroy R, Jamet A, Petua P, Robert R, Frat JP, Thille AW. High-flow nasal cannula oxygen therapy versus noninvasive ventilation in immunocompromised patients with acute respiratory failure: an observational cohort study. Ann Intensive Care. 2016 Dec;6(1):45.
