# Supplementary material for: A multi-mechanism approach reduces length of stay in the ICU for severe COVID-19 patients
Source: PLoS One. 2021 Jan 7;16(1):e0245025. doi: 10.1371/journal.pone.0245025 (PMC7790264; doi:10.1371/journal.pone.0245025)
Supplement: Attachment — Submitted filename: Rebuttal letter PLOS ONE - VanBuren.pdf [file pone.0245025.s003.pdf]

November 14, 2020

Yu Ru Kou, PhD
Academic Editor
PLOS ONE

RE: PONE-D-20-29696
A multi-mechanism approach reduces length of stay in the ICU for severe COVID-19 patients

Dear Dr. Kou,

Thank you for considering our manuscript for publication in PLOS ONE. We appreciate the thoughtful comments made be the reviewers, and we are resubmitting the manuscript with an effort to be responsive to all comments. Below we list reviewer comments (red) and our manuscript edits (blue).

Reviewer #1: The authors present original data on the use of a multiple mechanism therapeutic approach (MMA) on patients with severe COVID-19 hospitalized in ICU. The report that the MMA was assciated with a decreases in average ICU length of stay, thereby causing a relevant unload of the hospital workflow arounf COVID-19, which was a critical issue during the first outbreak.

Specific comments:

- the rapid outbreak of COVID-19 cases especially during the first breakout was responsible for a total derangement of healthcare services. One of the consequences was the generation of a strong selection bias on hospital admissions. In fact, hospital admission for Acute Myocardial Infarction and other acute cardiovascular diseases were dramaticaly reduced (Reduction of hospitalizations for myocardial infarction in Italy in the COVID-19 era. Eur Heart J. 2020;41(22):2083-2088. doi: 10.1093/eurheartj/ehaa409. - COVID-19 pandemic and admission rates for and management of acute coronary syndromes in England. Lancet. 2020;396(10248):381-389. doi: 10.1016/S0140-6736(20)31356-8.). Similarly, a selection bias was also suggested for COVID-19 patients, whereas the most severe cases probably did not make it to the hospital, which might then have caused an underestiation of death. Please comment on this issue;

RESPONSE: Yes we agree and we will make the following comments and cite the references in the manuscript:

Rapid outbreaks of COVID-19 cases are responsible for a total disruption of healthcare services. This was particularly true for early outbreaks, and selection bias in hospital admissions is one of the consequences of those circumstances. For example, hospital admissions for acute myocardial infarction and other acute cardiovascular diseases were dramatically reduced [citations]. We acknowledge that there could have been a selection bias in this study. However, the scope of our study addresses a treatment for critically-ill COVID-19 hospitalized patients in the ICU so therefore we do not believe that this selection bias affects the outcome of our study.

- the authors report IOT with mechanical ventilation being the only independent predictor of in-hospital death in this cohort. A recent analysis incuding over 75000 COVID-19 patients, of which 4344 were under intensive care found age, cardiovascular risk factors or comorbdities and CV complications were independent predictors of in-hospital death (Impact of cardiovascular risk profile on COVID-19 outcome. A meta-analysis. PLoS ONE 2020; 15(8): e0237131. https://doi.org/10.1371/journal.pone.0237131). Did the authors find similar results in their cohort? please discuss this aspect in the manuscript;

Mechanical ventilation was the strongest predictor of mortality in this cohort. However, age and cardiovascular complications have been identified as confounding factors [citation] and were used to adjust the logistic regression model for mortality.

- in this regard, the auhtors report "a strong relationship between treatment and age as predictors of LOS, showing greater benefit in reducing LOS in ICU for older patients". In light of this finding, how do the authors explain the lack of association between age and death? Might it be related with the limited sample size?

RESPONSE: The lack of association between age and death is likely due to the limited sample size in this study. We amend the manuscript as described in another response below.

- the authors state that "The database analyzed during the current study are available from the corresponding author on request.". HOwever, this doesn't comply to journal policies on data sharing. Please refer to authors' guidelines;

RESPONSE: We will make the data available as a supplementary file.

Reviewer #2: The authors assessed the impact of a "multiple mechanism therapeutic approach" (MMA) on the clinical management of patients with severe COVID-19. They found that the "MMA" approach was assciated with a decrease in length of stay in the ICU.

Comments:

- please, describe the criteria for selection of study centers. Please, also report how many centers were invited and the percentage of participating centers from those invited;

Our hospital selection was based on the availability of ICU units and a clinical lab that had Ferritin, D-dimer, PCR, and PCT testing. The hospitals in San Pedro Sula, Cortes, were selected because this the virus spread rapidly across this city. We did not invite other centers in Honduras, as they lack of materials or ICU units for COVID 19 patients.

- how were clinical endpoints reported? do the authors have information on thrombotic events?

Endpoints for patients were either death or discharge from the hospital. Information on thrombotic events were not captured for this study.

- despite many efforts, clinical information on female patients is still underrepresented compared to males. This issue has been evan larger with COVID-19. (Sabatino J. et al. Women's perspective on the COVID-19 pandemic: Walking into a post-peak phase. Int J Cardiol. 2020:S0167-5273(20)33552-X. doi: 10.1016/j.ijcard.2020.08.025.). Could the authors please report their results stratified by gender (e.g. in a summary table) and comment about eventual differences?

RESPONSE: A table that stratifies results by gender has been added to the manuscript.

There were 49 males and 19 females in the study. The survival rate for both males and females was 74% [S5 Table].

Given the the relatively small number of females in this study, we are not confident in providing further analysis of stratified data.

- lenght of stay is an obvious proxy of mortality, how did the authors managed the shorter LOS for early deaths? was any correction applied?

Limitations:

We examined mortality as a potential proxy for LOS and found no association between LOS and mortality. This is likely because of the small sample size in this study. Correcting for mortality (or even leaving all those patients who died out of the analysis) did not make a substantial difference in the LOS difference measured.

- a recent meta-analysis including over 4000 COVID-19 patients under intensive care identified age as an independent predictor of in-hospital death (Sabatino J. et al. Impact of cardiovascular risk profile on COVID-19 outcome. A meta-analysis. PLoS One. 2020;15(8):e0237131. doi: 10.1371/journal.pone.0237131.). How do the authors explain the lack of association in their cohort?

RESPONSE: yes we agree with the findings that age is an independent predictor of in-hospital death and will addend the manuscript as follows:

Limitations:

Age is a predictor of mortality in severe COVID-19 disease globally [cite paper above], but we did not identify an association between age and mortality in our study.The mean age in our study was 54 and our study population over 65 years of age was relatively small. The lack of association in our study between age and mortality is potentially explained by lower numbers of older people reporting to the hospital during the early stages of the pandemic, likely due to national policies requesting people to stay home unless severely ill.

- Did the authors find any association between cardiovascular comorbidities and in-hospital death?

RESPONSE: Yes we found the association between cardiovascular comorbidities and in-hospital death was found. We will amend the manuscript as follows:

Limitations:

Hypertension was a key confounder in the analysis and it was used to adjust for the regression of mortality. It was statistically significant in the univariant analysis. Thus, we expect that potential acute cardiovascular and thrombotic events contributed to mortality in both groups, although those events were not captured by our analysis.

Thank you for consideration of our manuscript for publication in PLOS ONE. We appreciate the time and effort that you and the reviewers have put in to making our publication stronger. Please call or email if you have any further questions or concerns.

Kind regards,

Vincent VanBuren, PhD
Texas A&M College of Medicine
vanburen@tamu.edu
512.970.2057
